# Reconfigurable Magnetic Nanopore Platform for Selective Trapping

**DOI:** 10.1002/smsc.70392

**Published:** 2026-09-08

**Authors:** Nageswar Reddy Sanamreddy, Jeanne Maunier, Malavika Kayyil Veedu, Anastasiia Sapunova, Nicolò Maccaferri, Denis Garoli, Jérôme Wenger, Paolo Vavassori

**Affiliations:** ^1^ CIC nanoGUNE BRTA Donostia‐San Sebastian Spain; ^2^ Polymers and Advanced Materials: Physics, Chemistry and Technology University of the Basque Country (UPV/EHU) Donostia‐San Sebastian Spain; ^3^ Aix Marseille Univ. CNRS Centrale Med Institut Fresnel AMUTech Marseille France; ^4^ Istituto Italiano di Tecnologia Genova Italy; ^5^ Ultrafast Nanoscience Group Department of Physics Umeå University Umeå Sweden; ^6^ Umeå Centre for Microbial Research Umeå University Umeå Sweden; ^7^ Wallenberg Initiative Materials Science for Sustainability Department of Physics Umeå University Umeå Sweden; ^8^ Dipartimento di Scienze e Metodi dell’Ingegneria Università degli Studi di Modena e Reggio Emilia Reggio Emilia Italy; ^9^ IKERBASQUE Basque Foundation for Science Bilbao Spain

**Keywords:** active control, fluorescence microscopy, magnetic nanoparticles, magnetic tweezers, nanopores, sensing, vortex state

## Abstract

Solid‐state nanopores offer a powerful platform for nanoscale analysis of individual analytes, including biomolecules and functionalized nanoparticles, by confining them within a precisely defined sensing region. However, their inherently passive operation restricts practical applications, as they cannot precisely control particle position or dynamics inside the pore. Here, we introduce magnetic nanopore architectures that integrate a ferromagnetic layer into the nanopore system. Acting as a magnetic discontinuity within an otherwise uniformly magnetized film, the nanopore generates localized stray magnetic fields that enable magnetic tweezing of magnetic nanoparticles, which can be functionalized with fluorescent biomolecules. Importantly, the nanopore geometry is designed to reversibly switch between a nearly uniform magnetization state and a magnetic flux‐closure state through the application of momentary magnetic fields of predetermined amplitude. This capability allows the magnetic tweezing effect to be selectively activated or deactivated, enabling controlled capture and release of tagged biomolecules on demand. As a proof of concept, we demonstrate the selective magnetic trapping of fluorescent magnetic particles. Our work establishes a robust and controllable approach for reconfigurable, on‐chip magnetic nanopore platforms capable of selective trapping and high‐throughput single‐particle detection.

## Graphical abstract

Reconfigurable magnetic nanopores enable reversible switching between trapping and non‐trapping states by controlling the magnetic configuration around the nanopore. This strategy provides selective localization of magnetic nanoparticles, opening new opportunities for programable nanopore sensing and active single‐particle manipulation.

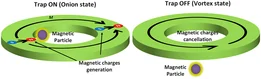

## 1 Introduction

Solid‐state nanopores are versatile single‐entity sensing platf‐orms where analytes or nanoparticles tagged with analytes are detected by means of electrical or optical readouts as they pass through a nanometer‐scale aperture [1, 2, 3, 4, 5]. Their mechanical stability, structural robustness, and tuneable geometry enable label‐free detection of single molecules through ionic current modulation and complementary optical modalities [1, 2, 4, 6, 7, 8, 9, 10, 11, 12, 13, 14, 15, 16]. Although active manipulation approaches such as optical and plasmonic trapping have been explored, robust and reconfigurable control of analyte localization within nanopore structures remains challenging [17, 18, 19, 20, 21, 22, 23, 24, 25, 26, 27, 28, 29, 30]. In fact, uncertainty in particle position and transient time within the pore limits robust signal extraction [1, 3, 4, 6, 8, 9, 10, 11, 12, 13, 14, 15, 16, 24, 25, 26, 27, 28, 29, 30]. Difficulty in localizing and maintaining analytes or nanoparticles tagged with analytes [31, 32, 33, 34, 35, 36, 37] within the sensing region for long enough time compromises signal integrity, reduces reproducibility, and limits advanced operations such as extended measurements and multi‐parameter analysis [1, 2, 3, 4, 6, 7, 8, 9, 10, 11, 12, 13, 14, 15, 16, 24, 25, 26, 27, 28, 29, 30]. Furthermore, recent reviews have highlighted the growing need for better analyte capture, longer residence time, and advanced signal processing to improve the performance of solid‐state nanopore sensing platforms [10, 14].

This work presents a magnetically reconfigurable nanopore platform that enables programable ON/OFF magnetic trapping through reversible magnetic‐state switching, addressing one of the key limitations of nanopore sensing. As a proof of concept, we demonstrate precise localization and retention of magnetic nanoparticles within the nanopore sensing region using remotely actuated magnetic nanotweezers. This platform is designed for analytes tagged with magnetic nanoparticles, which is a well‐established technique in biosensing and biomedicine. In this method, the magnetic nanoparticle acts as a tool, while the connected non‐magnetic analyte remains the sensing target. These magnetic nanoparticle labels have been widely used for biomolecular detection, targeted drug delivery, multimodal imaging, and theranostic applications, indicating the broad applicability of magnetic nanoparticle functionalisation [31, 32, 33, 34, 35, 36, 37].

Established methods such as focused ion beam (FIB) milling, transmission electron microscopy (TEM) drilling, and dielectric breakdown enable nanopore fabrication across a wide range of materials [24, 38, 39, 40, 41, 42, 43]. During the last decade, the incorporation of trapping/tweezing functionalities into such platforms has received significant attention [17, 18, 19, 20, 21, 22, 23, 24, 25, 26, 27, 28, 29, 30]. These efforts have primarily focused on improving structural precision, scalability, optical tweezing/signal enhancement, or electrical readout [17, 18, 19, 20, 21, 22, 23, 24, 25, 26, 27, 28, 29, 30, 38]. In contrast, approaches based on magnetic functionalities have been reported in few works [44, 45, 46], and magnetic tweezing within nanopore architectures has not yet been experimentally demonstrated.

**FIGURE 1:**
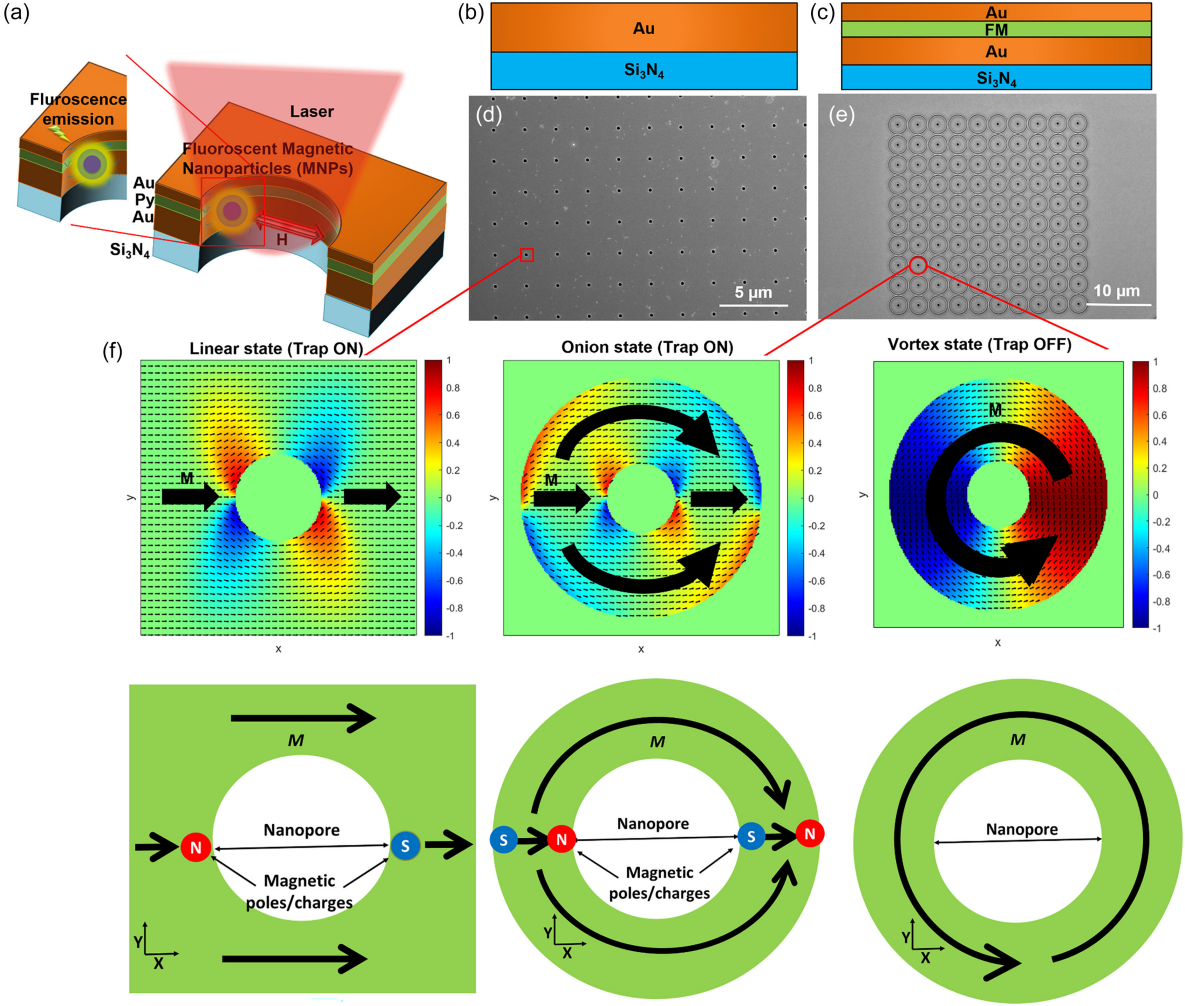
(a) Schematic illustration of the experimental setup and procedure. Studied multilayer systems: (b) Si_3_N_4_/Au/Ti and (c) Si_3_N_4_/Au/Py/Ti, deposited using an evaporator. SEM images of the periodic arrays of (d) nanopores and (b,c,e) circular trench surrounded nanopores fabricated by FIB milling on as‐deposited layers. (f) Magnetic configurations and corresponding schematic for the structures shown in (d) and (e), illustrating the linear, onion, and vortex magnetization states.

Here, we experimentally demonstrate how magnetic nanopores can be used to manipulate and detect individual nanoparticles (Figure 1a). According to a concept previously reported theoretically [44], the basic idea is to create a magnetic discontinuity by patterning a nanopore in an otherwise continuous magnetic thin film. This geometric configuration generates localized stray‐field gradients near the pore edges, acting as nanoscale magnetic tweezers that can effectively trap magnetic nanoparticles (MNPs), which can also be tagged with biomolecules to combine trapping and biosensing. Here, we developed a reconfigurable magnetic nanopore platform that allows for selective trapping of MNPs tagged with fluorescent biomolecules (hereafter termed fluorescent MNPs). The design features two reversible magnetic configurations: a nearly uniformly magnetized state (dubbed “onion” state in the literature) and a magnetic flux closure (called “vortex” state), which can be actively reconfigured by externally applied magnetic fields [47, 48, 49, 50, 51, 52, 53]. As illustrated in Figure 1, the onion state (trap ON) produces magnetic charges (poles) at the edge of the pores, each one creating magnetic stray‐field gradients which can be used for trapping MNPs. Additionally, the vortex state (trap OFF) establishes a flux‐closure configuration that minimizes stray fields, thus removing the trapping capability. This switching between magnetic states acts as a selective ON/OFF mechanism for the active trapping of fluorescent MNPs within the nanopore. This approach provides the groundwork for a fully adaptable nanopore system, enabling the deterministic capture of nanoscale objects on demand. Our platform represents a significant step beyond passive nanopore sensing by introducing programable magnetic trapping within solid‐state nanopore technology. This work demonstrates the feasibility of a magnetically reconfigurable trapping platform as a prerequisite for future active nanopore sensing and sequencing applications.

## 2 Materials and Methods

### 2.1 Sample Nanofabrication

We employed a standard fabrication process to create silicon nitride (Si_3_N_4_) membranes [54, 55, 56]. To ensure the mechanical integrity required for magnetic nanopore applications, we utilized a 500 μm thick silicon substrate that was coated on both sides with 100 nm of Si_3_N_4_ to fabricate suspended membranes (see Figure S1 for a schematic of the membrane fabrication process). Then, multilayer metallic films were deposited onto the suspended membranes. Two configurations were prepared: a non‐magnetic reference system comprising Gold ‐ Au (100 nm)/ Titanium ‐ Ti (5 nm) (Figure 1b), and with a magnetic system comprising Au (100 nm)/ Ferromagnetic (FM) layer: Permalloy ‐ Py, Ni_80_Fe_20_ (20 nm)/Ti (5 nm), (Figure 1c). We started the deposition with 5 nm Ti layer to enhance the adhesion of the multilayer onto the Si_3_N_4_ surface. The 5 nm‐thick Ti capping layer acts as a protective barrier against oxidation for the FM layer. Also, in this study, Ti was used as the capping layer because it produces a significantly lower fluorescence background, making it more suitable for the fluorescence‐based measurements reported below. Further, we used these substrates to fabricate periodic arrays of nanopores by means of FIB milling, as illustrated in Figure 1d. An additional configuration of the nanopores array involved the patterning of a circular trench, completely engraved through the metallic layers, concentric with the individual nanopores, thereby defining a circular FM region (hereafter referred to as circular FM structures, Figure 1e). This design was chosen to stabilize different remanent magnetic configurations, specifically onion and vortex states, allowing for switchable ON/OFF trapping capabilities.

### 2.2 MOKE Microscopy

Magneto‐optical Kerr effect (MOKE) microscopy measurements to record hysteresis loops were performed using a commercial system (Evico Magnetics). The magnetic response of the nanopore structures was characterized in the longitudinal Kerr geometry by monitoring the polarization rotation of the reflected light. An in‐plane magnetic field was applied to control the magnetization states.

### 2.3 Fluorescent Nanoparticles

Commercially available conjugated polymer nanoparticles incorporating iron oxide (fluorescent MNPs) were used as received (Merck 905038‐250UL). Absorbance and fluorescence emission spectra were recorded on a Tecan Spark 10 M spectrofluorometer (Figure S2). Fluorescence correlation spectroscopy was performed to characterize the nanoparticle size, resulting in a nanoparticle diameter of approximately 90 nm (Figure S3), in good agreement with the manufacturer specifications. The nanoparticle concentration of the stock solution was determined to be 0.6 nM or 3.6 × 10^11^ nanoparticles/mL.

### 2.4 Optical Fluorescence Microscope

A custom‐built confocal microscope operates with a pulsed laser (Toptica TVIS, 4 ps duration, 40 MHz repetition rate, operational wavelength 490 nm) with 1.5 µW power coupled to the microscope. The objective used was a Zeiss C Apochromat 63x 1.2 NA with water immersion. A 50 µm pinhole was conjugated to the sample plane. The detection was performed with a Picoquant MPD avalanche photodiode connected to a Hydraharp4 time‐resolved counting electronic module (Picoquant). The detection integrates the fluorescence signal over the 500–550 and 570–600 nm regions. A 3D piezoelectric stage was used to scan the sample, with 15 ms integration time per pixel and 50× 50‐pixels images.

## 3 Results and Discussion

Two nanopore designs were investigated. (i) SAMPLE‐A, periodic square nanopore arrays (250 nm pore diameter, 2.5 µm pitch), were studied in both non‐magnetic (reference) and magnetic configurations as described above (see Figure 1d); (ii) SAMPLE B, where circular FM structures interrupt the continuous FM layer to form outer annular ring of diameter 1–2.5 µm, was examined only in the magnetic system (see Figure 1e).

**FIGURE 2:**
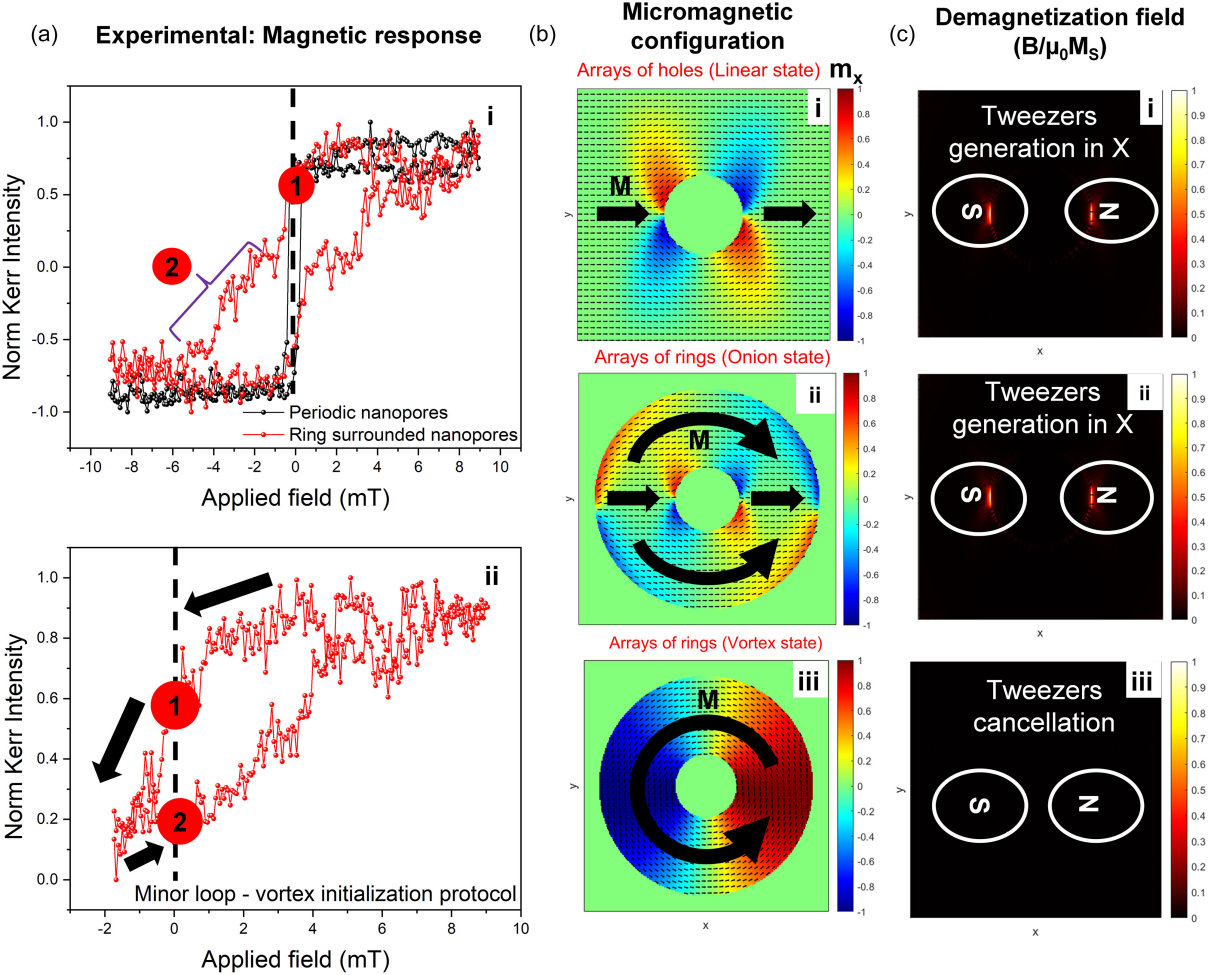
(a) Magnetic response of periodic nanopores and circular FM structures measured by MOKE microscopy, showing (i) the full hysteresis loop and (ii) the vortex loop highlighting the demagnetization protocol for the circular FM structures. Point 1 corresponds to the remanent Linear state of the periodic nanopores and the onion state of the circular FM structures, whereas point 2 corresponds to the vortex state of the circular FM structures. (b) Micromagnetic simulations showing the remanent magnetization configurations corresponding to the linear (i), onion (ii), and vortex states (iii). (c) Corresponding demagnetization field for the magnetization states shown in (b).

Initially, we characterized the magnetic properties of SAMPLE‐A with the Py layer, using longitudinal MOKE microscopy. The hysteresis loop is reported in Figure 2a(i). It shows that an external magnetic field of approximately *H*  ≈ 4 mT is enough to saturate the array. The nearly square hysteresis loop (see Figure S4) indicates high remanence of the film and stable magnetization state after field removal. We supported this experimental finding with micromagnetic simulations performed using MuMax3 [57]. The simulated magnetization configuration after applying the field in *x* direction and relaxing to the remanent state exhibits a nearly uniform magnetic state (Figure 2b(i)), while the corresponding demagnetization field Figure 2c(i) reveals a strong field localization at the pore edges. These regions indicate the generation of magnetic surface charges where magnetization is nearly perpendicular to the pore boundary, generating localized stray field gradients which enable the creation of confined trapping sites. Furthermore, these trapping sites can be moved around the pore edge by changing the direction of the applied field (see Figure S5 for *y*‐oriented magnetization induced by an external magnetic field *H*_y_). The remanent state remains stable after removal of the applied field, preserving the localized stray‐field gradients at the pore edges.

Magnetic nanoparticles experience a force proportional to the gradient of the magnetic field magnitude, enabling trapping in regions where ∇∣*H*∣ is maximized. Because the magnetization state of the film, including the nanopore determines the spatial distribution of magnetic charges, a specific location of trapping hotspots along the circumference of the pore can be tuned by applying an external magnetic field along a specific direction. In this way, the external field acts as a control parameter for nanoscale trapping functionality.

Magnetic field gradients were computed using the demagnetization field output. The resulting two‐dimensional maps reported in Figure S6 reveal intense gradients localized at the nanopore edges, reaching magnitudes on the order of 10^8^ T/m. These exceptionally high gradients confirm the capability of the nanopore edges to function as strong and spatially confined magnetic trapping sites. Since the magnetic pore remains saturated even after removing the external magnetic field, this type of nanopore system is advantageous for stable trapping. However, for applications explored in this work, it would be desirable to implement a deterministic ON/OFF (SAMPLE B) capture of fluorescent MNPs. To address this limitation, we extend the nanopore geometry by fabricating circular FM structures. In such ring geometries, two characteristic magnetization configurations can be stabilized: the onion state, in which the magnetization follows the ring perimeter with the formation of two domain walls, and the vortex state, where the magnetization curls continuously, resulting in flux closure as schematically illustrated in Figure 1f. Figures 2a(i) (in red) and S7 depict the experimentally measured magnetic response of the circular FM rings obtained by MOKE microscopy. The full hysteresis loop in Figure 2a(i) (in red) exhibits a step‐like behavior, demonstrating the presence of intermediate magnetic states. The vortex loop in Figure 2a(ii) displays the demagnetization process for transitioning between trapping and non‐trapping states. This result is also supported by micromagnetic simulations (see Figure 2b(ii)), which shows the onion configuration, and the associated demagnetizing field (see Figure 2c(ii)). These reveal localized stray‐field gradients that generate the tweezers (i.e., switches ON) responsible for trapping. Figure 2b(iii) shows the vortex‐state magnetization configuration and its associated demagnetizing field is reported in Figure 2c(iii). In this case, the curled magnetization generates a flux closure configuration in which no magnetic charges are present at the nanopore edge, thereby switching off the tweezing effect. The magnetic trapping mechanism is governed by the stray‐field gradients generated by magnetic charges at the nanopore edge. In the onion state, magnetization terminates at the nanopore boundary, resulting in magnetic surface charges that generate strong localized stray fields and large gradients. Magnetic nanoparticles are drawn toward these high‐gradient regions, resulting in stable trapping at the nanopore edge. In contrast, the vortex state creates a closed magnetic flux configuration that removes magnetic surface charges at the nanopore edge, turning off the magnetic tweezing forces. Notably, the magnetic configuration around the nanopore can be toggled between onion and vortex states using an external field thanks to the engineered geometry of the nanopore (as discussed and shown in Figure 2).

**FIGURE 3:**
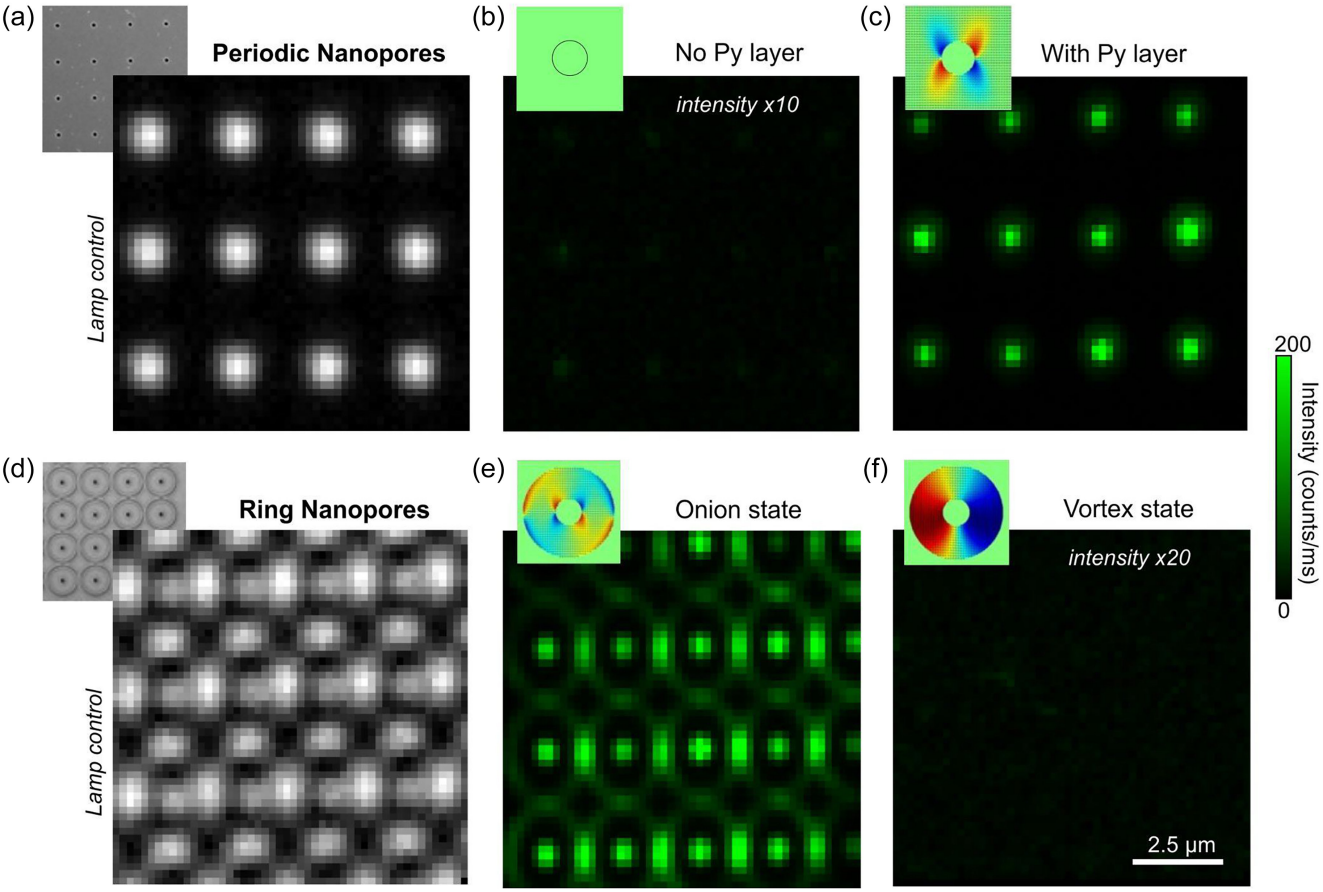
(a–c) Fluorescence characterization of the magnetic trapping for the periodic nanopores and (d–f) the ring‐surrounded nanopores. The transmission images in (a,d) with lamp illumination confirm the sample is well positioned at the microscope focus. All the images share the same 10 µm horizontal field of view. The fluorescence images in (b,c) and (e,f) are recorded with confocal laser scanning. In the absence of permalloy layer (b) and for the ring nanopores prepared in the vortex state (f), the contrast has been increased by 10 and 20× respectively, yet no fluorescence signal is visible from the residual luminescence background.

After developing and characterizing the reconfigurable magnetic nanopore platform, we evaluated its functionality using fluorescent MNPs. All nanopore samples were incubated for 15 min in a 0.6 nM stock solution of fluorescent MNPs, with pure water on the opposite side to facilitate translocation by diffusion. After incubation, the samples were thoroughly rinsed with ultrapure water and immediately characterized using fluorescence microscopy. For each sample, a brightfield transmission image (acquired using the microscope lamp) was recorded prior to fluorescence imaging to confirm proper sample positioning and focus (Figure 3a,d).

In the absence of nanoparticles, only weak residual background fluorescence was detected from the nanopore sample (see Figure S8). As a negative control, we incubated the SAMPLE‐A without the Py layer, with the same nanoparticles. As can be observed in Figure 3b, in this case no signal beyond the background fluorescence can be observed. The optical characterizations of the SAMPLE‐A with the Py layer resulted in periodic arrays of bright fluorescence spots with a 2.5 µm periodicity, corresponding to the nanopore positions (see Figure 3c). The fact that trapping is observed only in presence of the permalloy layer confirms the magnetic origin of the trapping mechanism. These experimental findings are highly consistent with micromagnetic simulations, confirming that nanoparticle trapping is governed by localized stray‐field gradients generated by the onion state and suppressed in the vortex state.

To verify that the observed bright spots originate from nanoparticle fluorescence, a bleaching experiment was performed by focusing the laser beam on a single nanopore (see Figure S9). The exponential decay of fluorescence intensity in the presence of the Py layer confirmed that the signal arose from nanoparticle bleaching, whereas the faint background luminescence exhibited 2 orders of magnitude weaker intensities.

Similar to the periodic nanopore array (SAMPLE‐A), fluorescence‐based experiments were performed on the circular FM structures (SAMPLE‐B, see Figure 3d–f) to evaluate the tweezing effect of nanopore corresponding to a specific magnetic configuration. When SAMPLE‐B was prepared in the onion state, we could measure a pronounced fluorescence signal localized around the nanopore region (Figure 3e), indicating the trapping of magnetic nanoparticles. This behavior is consistent with the presence of localized stray‐field gradients associated with the onion configuration, which generate effective magnetic trapping sites. In contrast, when SAMPLE‐B was prepared in the vortex state, i.e., in the absence of tweezing effect, no detectable fluorescence signal was observed in the vicinity of the nanopore (see Figure 3f). This can be attributed to the flux closure nature of the vortex configuration, which significantly reduces stray fields and associated field gradients; thus, MNPs could not be stably trapped and measured.

The clear difference in fluorescence response between these two states demonstrates that nanoparticle trapping is directly governed by the magnetic configuration of the nanopore. Importantly, since these configurations can be accessed through stabilization of magnetic field protocols, the system enables selective trapping. These results establish a direct experimental link between ON/OFF magnetic states and nanoparticle trapping, highlighting the functionality of the platform as a reconfigurable magnetic trapping system. These findings demonstrate the feasibility of the proposed magnetically reconfigurable trapping concept to implement a programable ON/OFF localization of fluorescent MNPs at nanopores, laying the groundwork for future active nanopore sensing applications.

**FIGURE 4:**
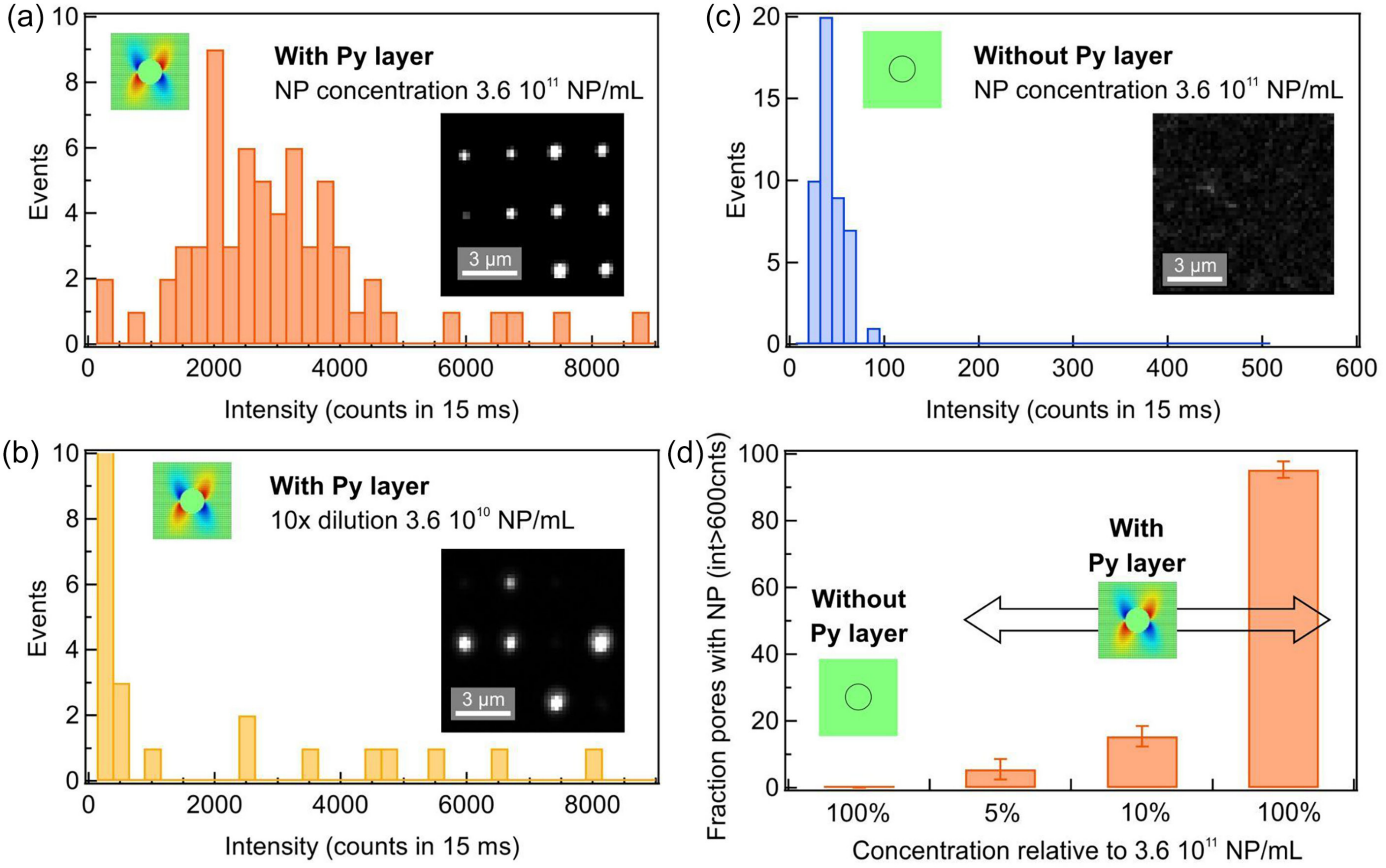
(a–c) Distribution of the fluorescence intensities recorded for single nanopore in presence (a,b) or absence (c) of the Py layer. (d) Fraction of nanopores containing fluorescent NPs (defined using an intensity threshold of 600 counts/15 ms) for different concentrations of the NP solution.

Finally, the fluorescence intensity distributions of SAMPLE‐A with the Py layer, as shown in Figure 4a,b (and without Py in Figure 4c), were analyzed at varying nanoparticle concentrations. At a stock concentration of 3.6 × 10^11^ NPs/mL, over 95% of nanopores exhibited characteristic fluorescence signals, indicating successful trapping (potentially of multiple fluorescent MNPs). A tenfold reduction in concentration decreased the fraction of loaded nanopores to ~15%, and a 20‐fold dilution further reduced this to ~6% (see Figures 4 and S10). This concentration dependence provides additional evidence for the effective magnetic trapping of fluorescent MNPs by the nanopore array.

## 4 Conclusion and Outlook

This study demonstrates a reconfigurable magnetic nanopore platform enabling switchable ON/OFF tweezing functionality via programable magnetization states by taking advantage of the change from a trap (near‐uniform) state to a non‐trap (vortex) state. Fluorescence‐based measurements verify the selective trapping of magnetic nanoparticles. The platform’s adaptability is highlighted by the coupling of optical readout and magnetic tweezing, which connects active trapping of nanoscale objects with nanopore sensing. The present work establishes the feasibility of programable magnetic trapping at nanopores as a proof of concept, providing a foundation for future studies on active nanopore sensing. Such integration aligns with the broader development of multifunctional nanopore systems, where electrical and optical modalities can be combined for enhanced sensitivity. The proven capacity to flip between trapping states opens new possibilities for nanopore device programmability. The approach introduced in this work is important for applications of nanopores in single‐particle analysis, biosensing, and nanofluidics that need precisely specified particle location and interaction within the sensing region. The utilization of magnetic structures provides a scalable and robust method in contrast to traditional trapping techniques.

The potential of intelligent nanopore sensing systems has recently been demonstrated through the integration of artificial intelligence (AI) and machine learning with nanopore tweezers for automated signal analysis and molecular discrimination [58]. Although AI‐powered data analysis represents an important direction for future nanopore sensing, the objective of the present work is to improve the physical reliability of nanopore measurements by enabling programable magnetic trapping and localization of analytes within the nanopore sensing region. This could improve signal stability and provide higher‐quality data for future AI‐assisted nanopore sensing.

## Author Contributions

N.R.S. developed the fabrication process, performed the magnetic measurements, carried out the micromagnetic simulations. J.M., M.K.V., and J.W. integrated the system and performed the particle trapping experiments. A.S. fabricated the Silicon nitride membranes. D.G. provided the funding and contributed to scientific discussions. N.M. and P.V. conceived the idea. P.V. supervised the work.

## Funding

This work was supported by the Marie Skłodowska‐Curie Actions Doctoral Network Project ‐ DYNAMO (Grant number 101072818); Agence Nationale de la Recherche under France 2030 Initiative (Grant number ANR‐24‐PEXM‐0001), Swedish Research Council (Grant number 2021‐05784 and number 2025‐04734), the Knut and Alice Wallenberg Foundation (Grant number 2023.0089), and the European Research Council (Grant number 101116253).

## Conflicts of Interest

The authors declare no conflicts of interest.

## Supporting information

(1) Schematic of suspended silicon nitride membrane fabrication process (2) Absorption and emission spectra of the nanoparticles. (3)  Fluorescence correlation spectroscopy data. (4). Magnetic response of periodic nanopores (5 & 6). Micromagnetic simulations of permalloy (7). Magnetic response of rings with varying diameters (8) Fluorescence Imaging the nanopores in absence of fluorescent nanoparticles (9) Fluorescence intensity time traces (10) Trapping with different nanoparticle concentrations.

## Data Availability

The data that support the findings of this study are available from the corresponding author upon reasonable request.
