# Supplementary material for: Reconfigurable Magnetic Nanopore Platform for Selective Trapping
Source: Small Sci. 2026 Sep 8;6(9):e70392. doi: 10.1002/smsc.70392 (PMC13574000; doi:10.1002/smsc.70392)
Supplement: Supplementary file 1 [file SMSC-6-e70392-s001.pdf]

Supporting Information

Reconfigurable Magnetic Nanopore Platform for Selective Trapping

*Nageswar Reddy Sanamreddy,[1,2] Jeanne Maunier,[3] Malavika Kayyil Veedu,[3] Anastasiia Sapunova,[4] Nicolò Maccaferri,[5,6,7] Denis Garoli,[4,8] Jérôme Wenger,[3] Paolo Vavassori[1,9]**

[1]CIC nanoGUNE BRTA, Tolosa Hiribidea, 76, E-20018 Donostia-San Sebastian, Spain

[2]Polymers and Advanced Materials: Physics, Chemistry and Technology, University of the Basque Country (UPV/EHU), E-20018 Donostia-San Sebastian, Spain

[3]Aix Marseille Univ, CNRS, Centrale Med, Institut Fresnel, AMUTech, 13013 Marseille, France

[4]Istituto Italiano di Tecnologia, Via Morego 30, Genova, Italy

[5]Ultrafast Nanoscience Group, Department of Physics, Umeå University, Linnaeus väg 24, 901 87 Umeå, Sweden

[6]Umeå Centre for Microbial Research, Umeå University, 901 87 Umeå, Sweden

[6]Wallenberg Initiative Materials Science for Sustainability, Department of Physics, Umeå University, Linnaeus väg 24, 901 87 Umeå, Sweden

[8]Dipartimento di Scienze e Metodi dell'Ingegneria, Università degli Studi di Modena e Reggio Emilia, Via Amendola 2, 43122, Reggio Emilia, Italy

[9]IKERBASQUE, Basque Foundation for Science, Plaza Euskadi, 5, E-48009 Bilbao, Spain

Email: n.sanamreddy@gmail.com, jnn.maunier@gmail.com, malavikaiiserb@gmail.com, Anastasiia.Sapunova@iit.it, nicolo.maccaferri@umu.se, denis.garoli@iit.it, jerome.wenger@fresnel.fr

Corresponding author *: p.vavassori@nanogune.eu

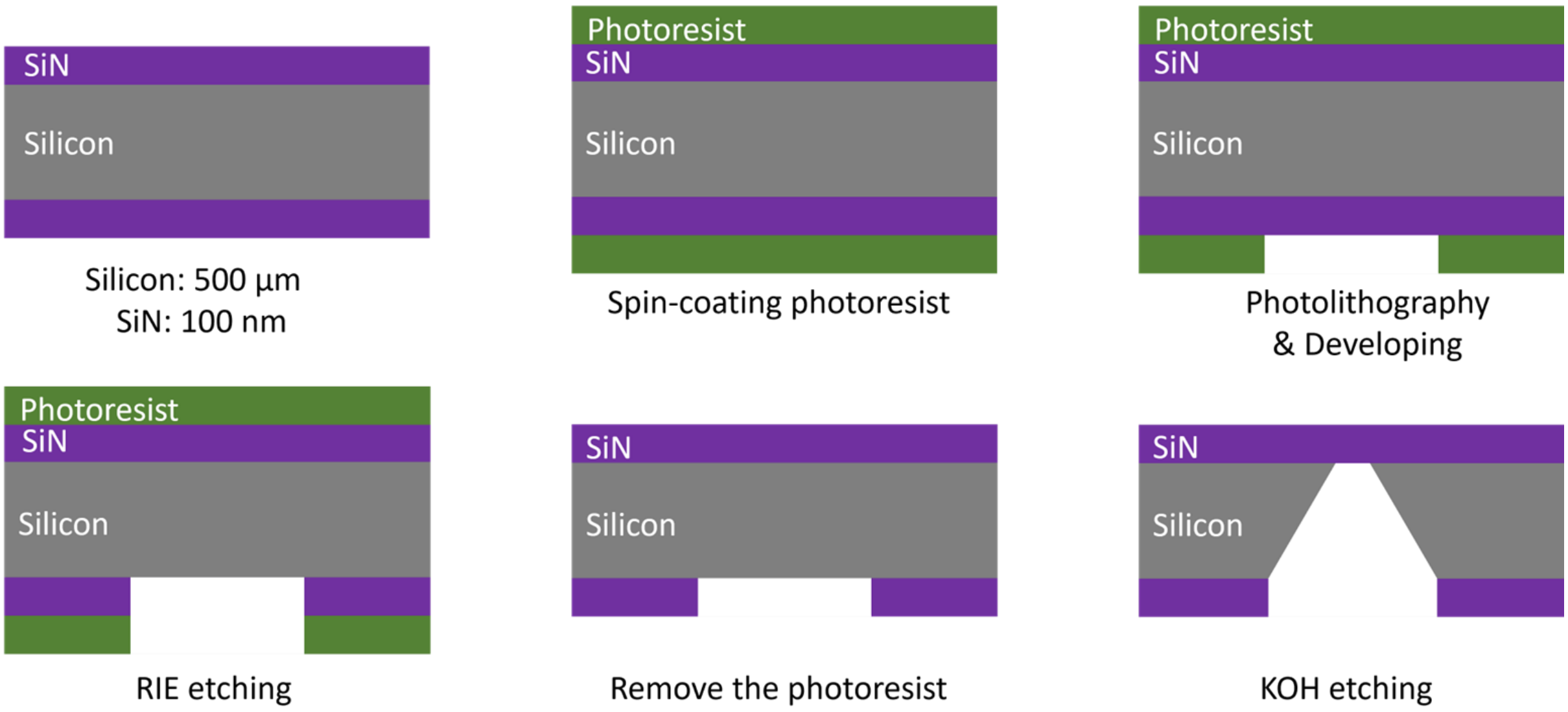

Figure S1. Schematic of suspended Silicon nitride (Si_3N_4) mesmbrane fabrication process

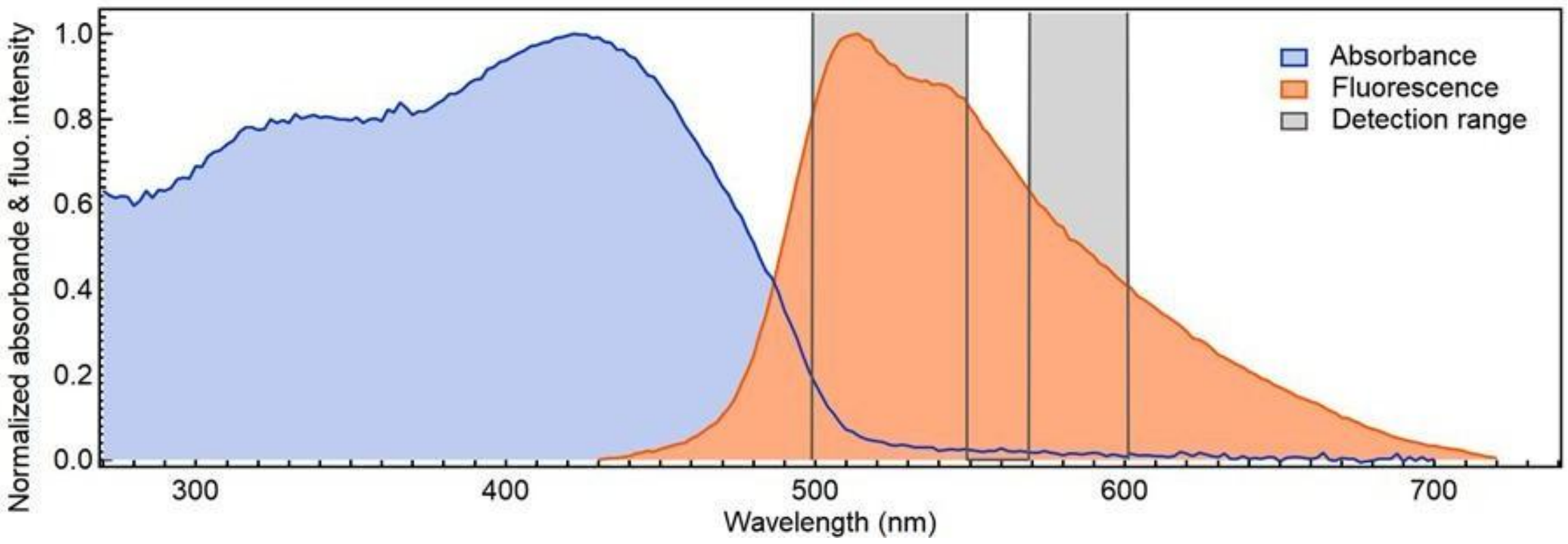

Figure S2. Absorption and emission spectra of the nanoparticles containing iron oxide. In the optical fluorescence microscopy experiments, the excitation is performed at 490 nm while the detection uses the bands 500-550 and 570-600 nm.

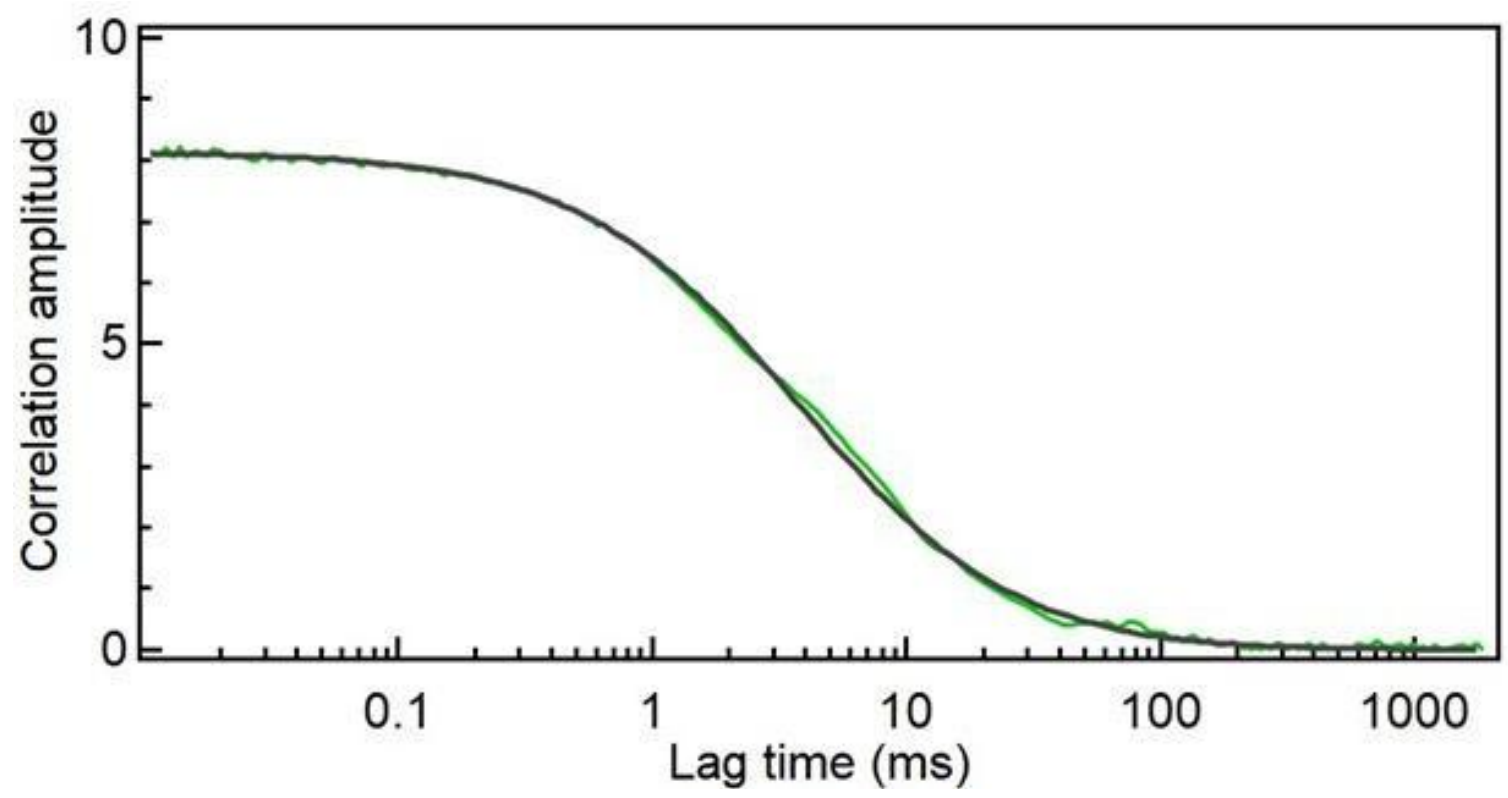

Figure S3 Fluorescence correlation spectroscopy (FCS) is performed to characterize the nanoparticle concentration and their diffusion coefficient and hydrodynamic radius. A 3.8 ms diffusion time is found which on our instrument corresponds to a nanoparticle diameter of 90 nm, in good agreement with the manufacturer specifications.

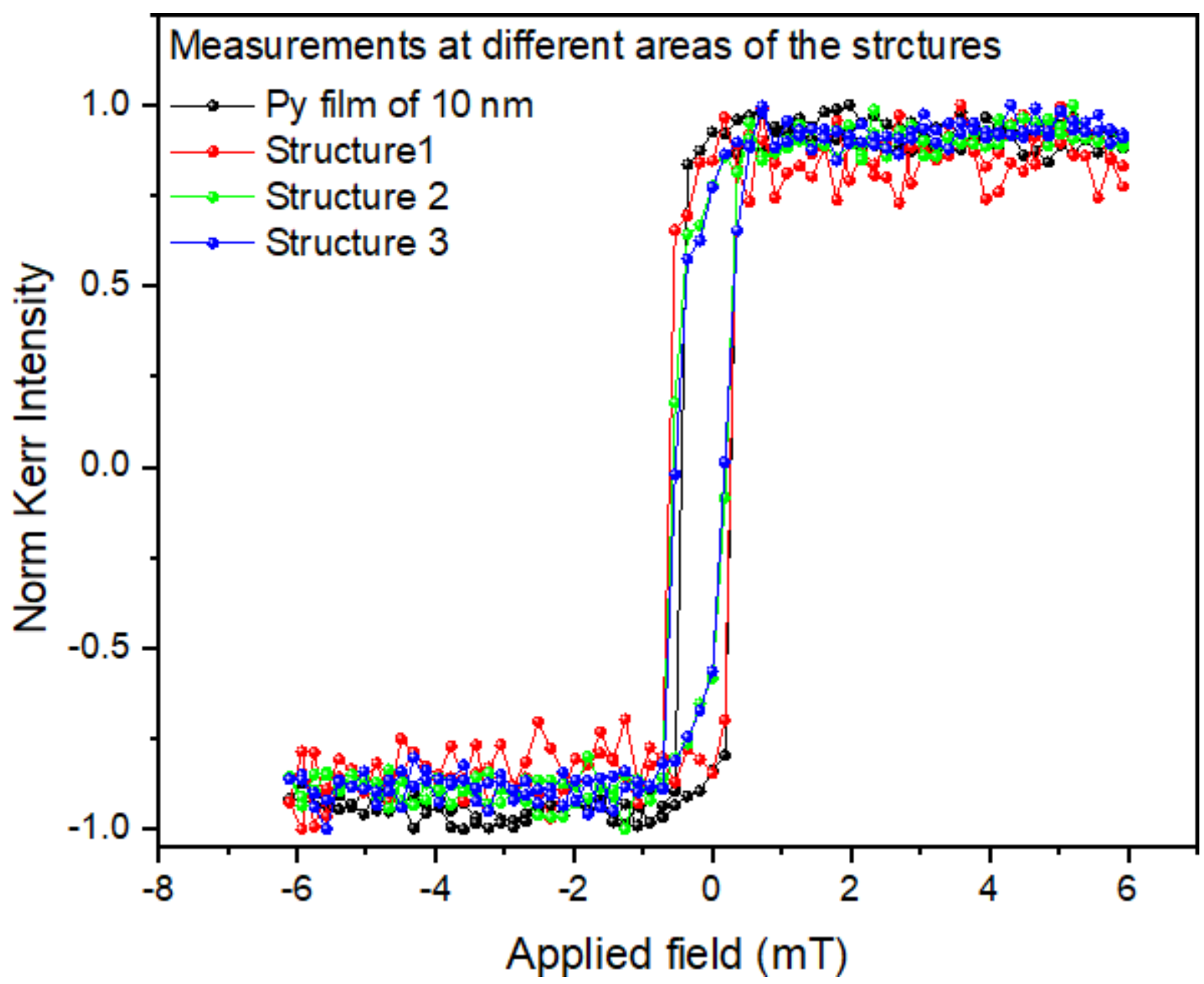

Figure S4. Magnetic response of periodic nanopores (SAMPLE-A) measured by MOKE microscopy on different areas fabricated by FIB milling on Au(100 nm)/Py(10 nm)/Ti(5 nm)

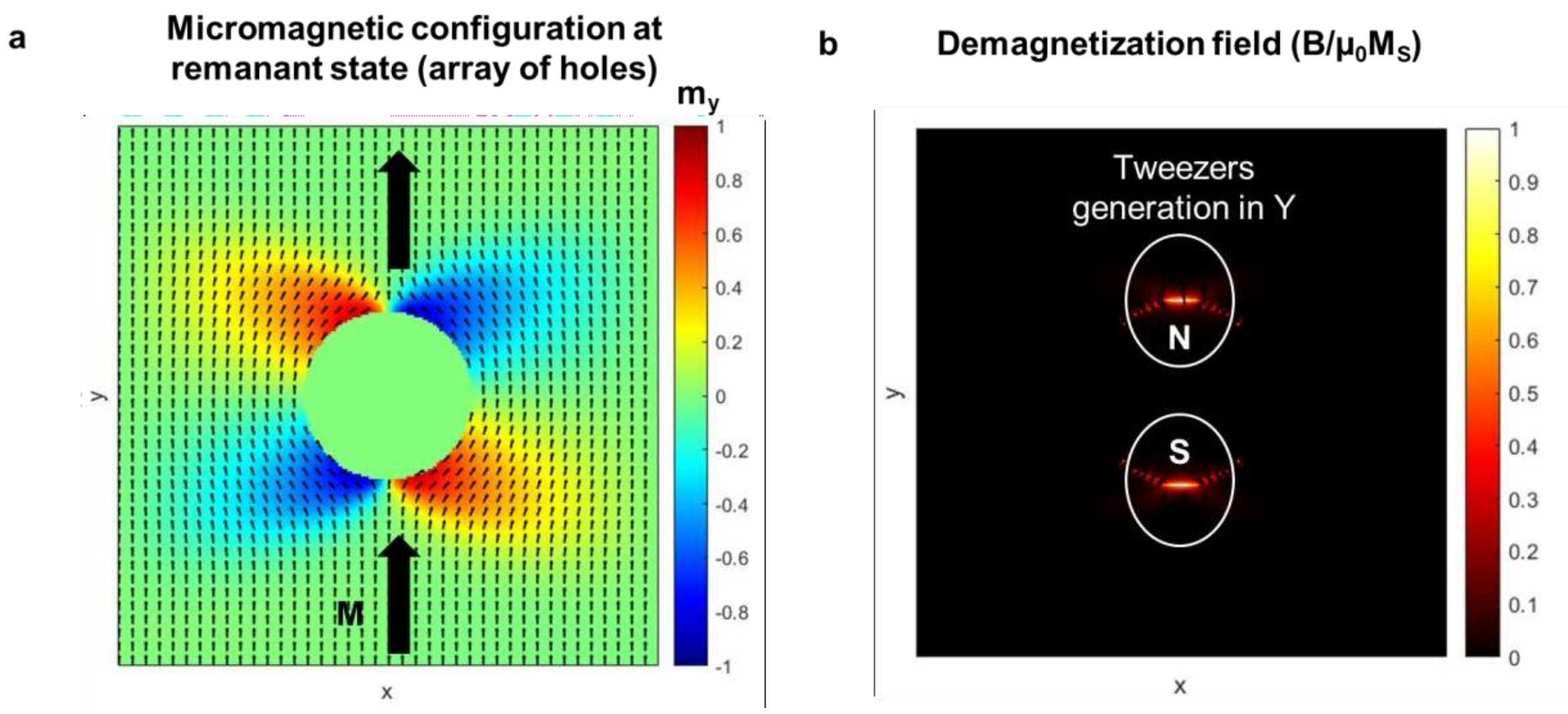

Figure S5. Micromagnetic simulations of permalloy after saturation and relaxation to remanence, showing (a) the magnetization configuration in Y direction, (b) corresponding demagnetization field.

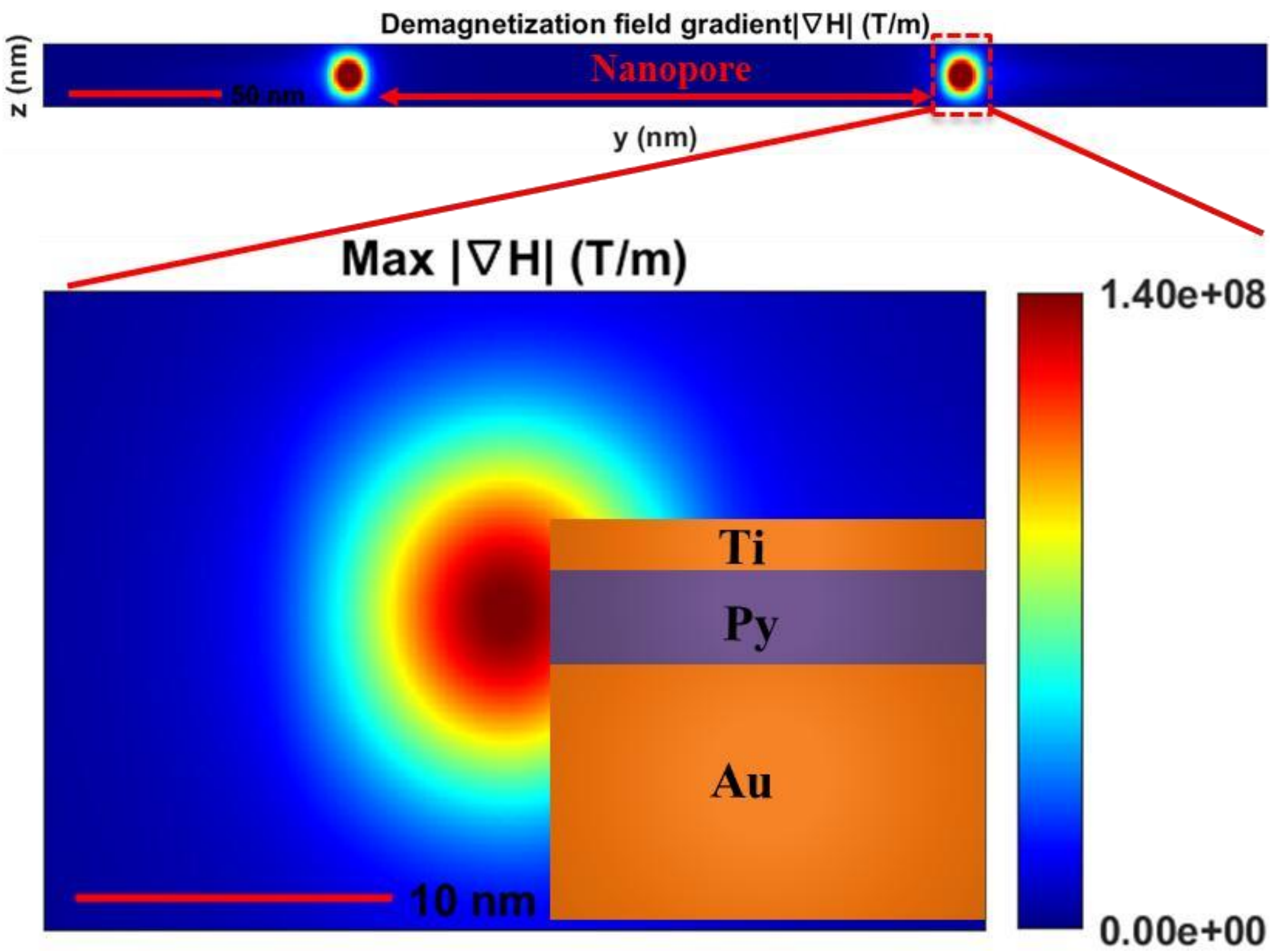

Figure S6. Micromagnetic simulations of permalloy showing the corresponding magnetic field gradient near the pore at remanent state

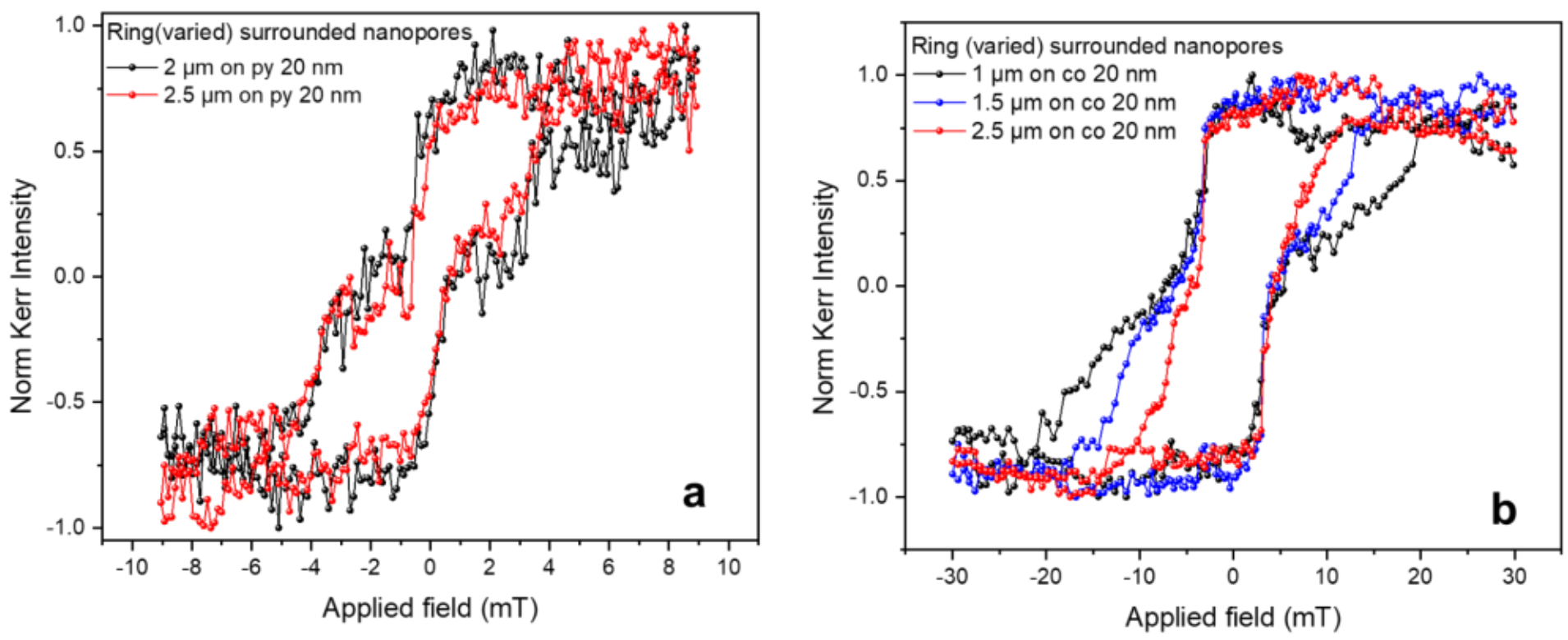

Figure S7. Magnetic response of SAMPLE-B fabricated on (a) Au (100 nm)/Py (20 nm)/Ti (5 nm) (b) Au (100 nm)/cobalt - Co(20 nm)/Ti (5 nm) , measured by MOKE microscopy.

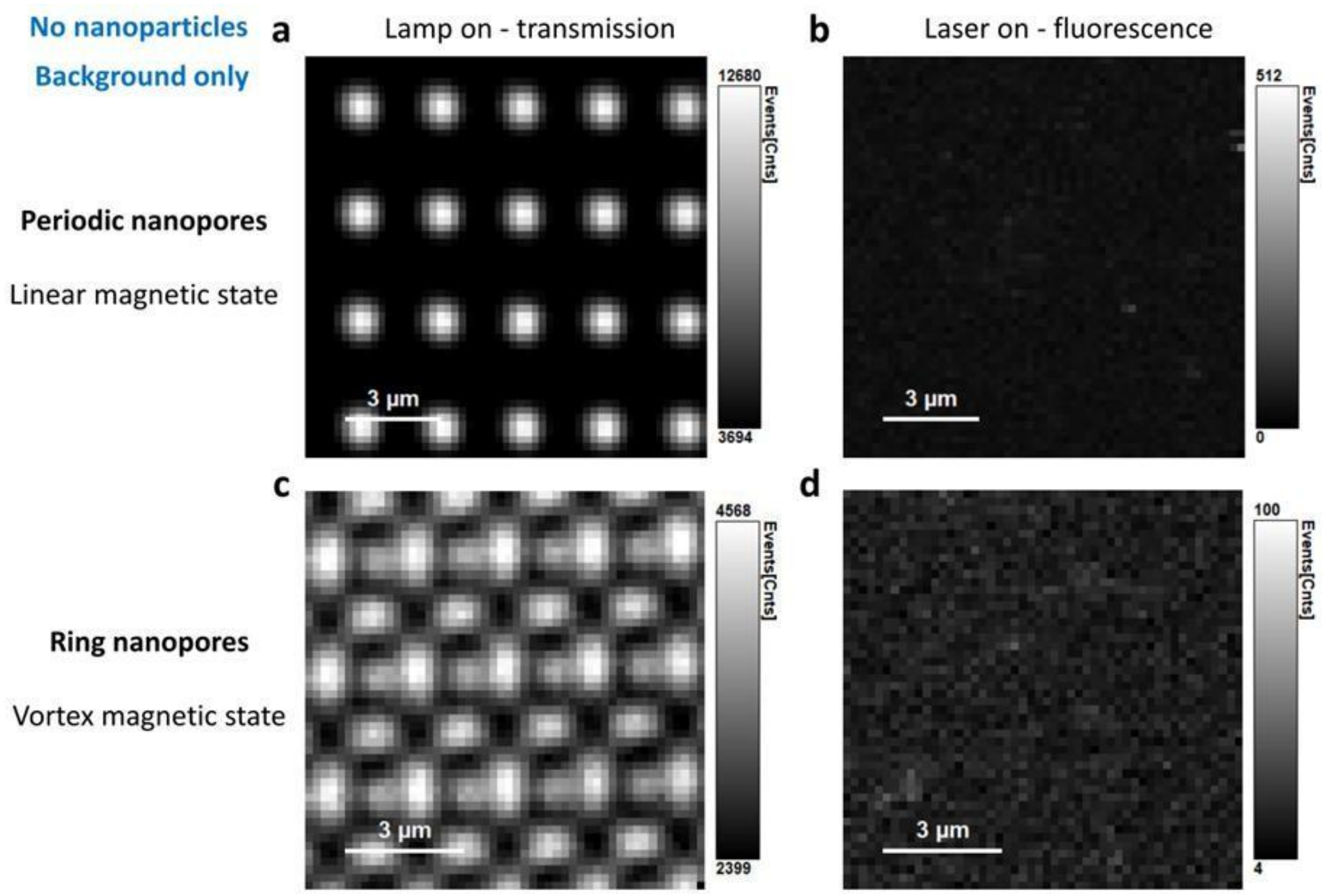

Figure S8. **Imaging the nanopores in absence of fluorescent nanoparticles**. Reference images of the sample with the lamp to localize the nanopores (a,c) and with the laser to excite the residual background luminescence (b,d). Laser power was 1.5 µW. Images (a,b) were recorded on the periodic nanopore array with the Py layer, while (c,d) correspond to the ring nanopore array configured in the vortex magnetic state. Confocal scanning with the microscope lamp ensures the sample is well positioned and at focus (a,c). In the same conditions, when the laser is used instead of the halogen lamp, only a weak

residual background luminescence is detected from the sample (b,d). Here the Ti blocking layer blocks efficiently the luminescence from the gold and Py layers.

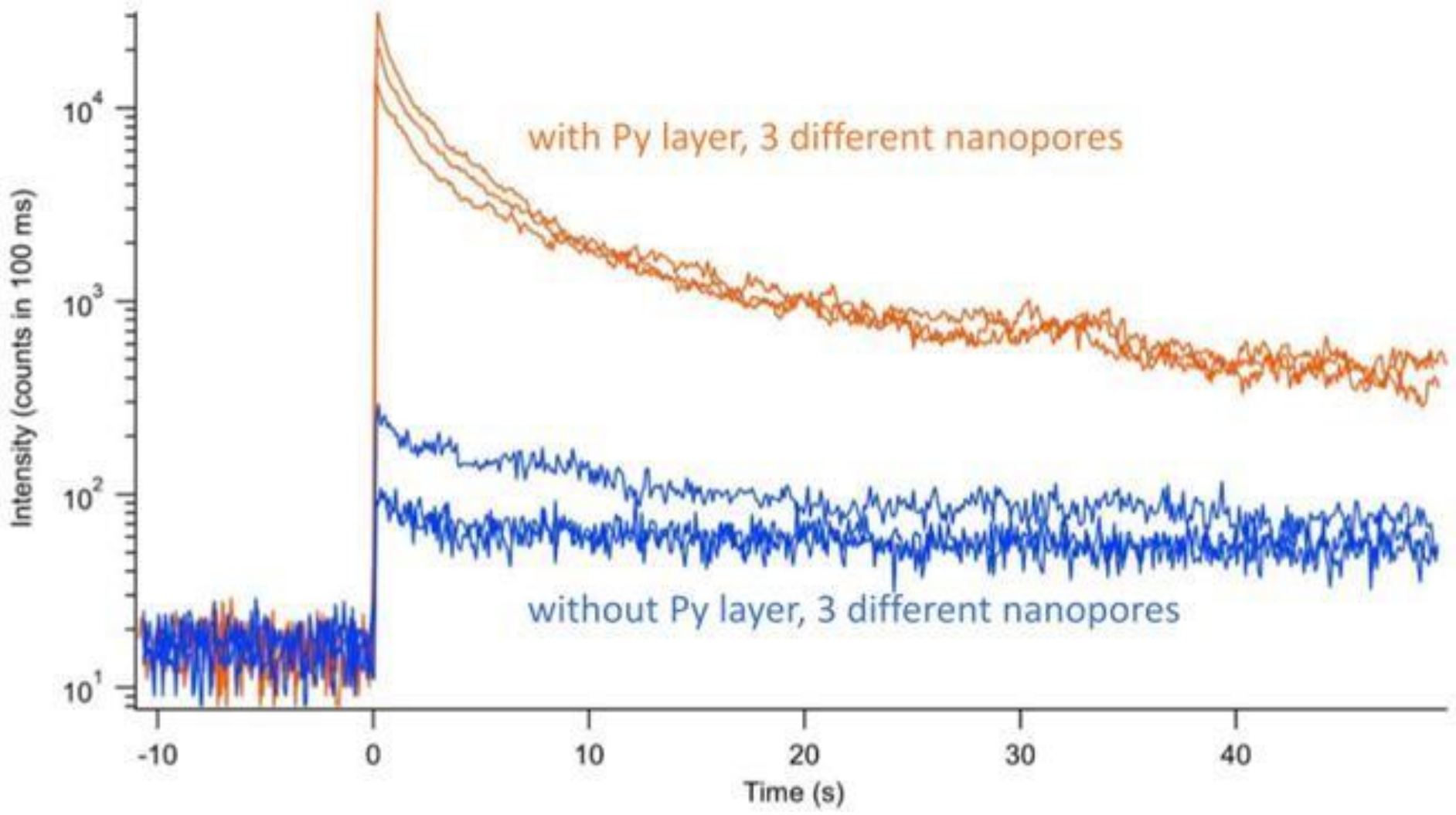

Figure S9. Fluorescence intensity time traces in presence and absence of the Py layer. The 1.5 µW laser is sent on the nanopores at t=0.

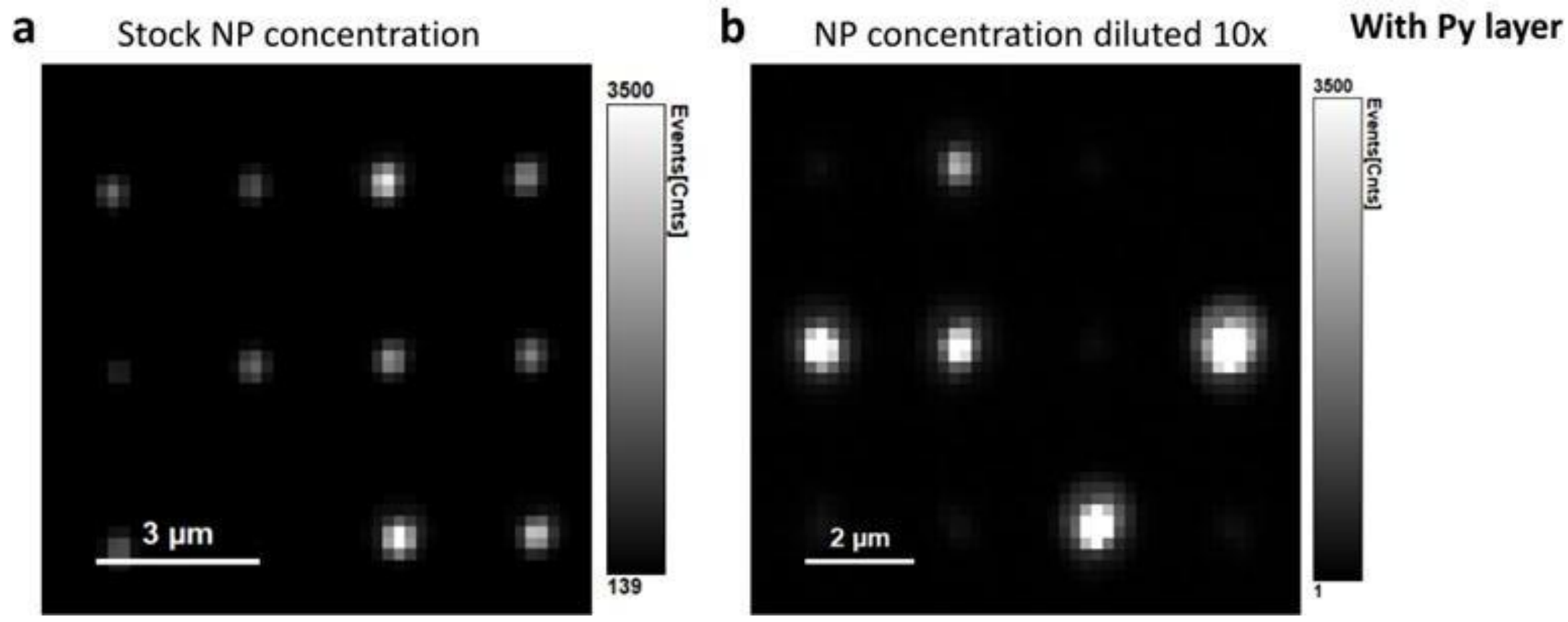

Figure S10. Fluorescence images of the nanopore array in presence of the Py layer allowing trapping with different concentrations of the NP solution. Lower NP concentration yields less nanopores with a trapped NP, confirming the trapping of fluorescent MNPs.

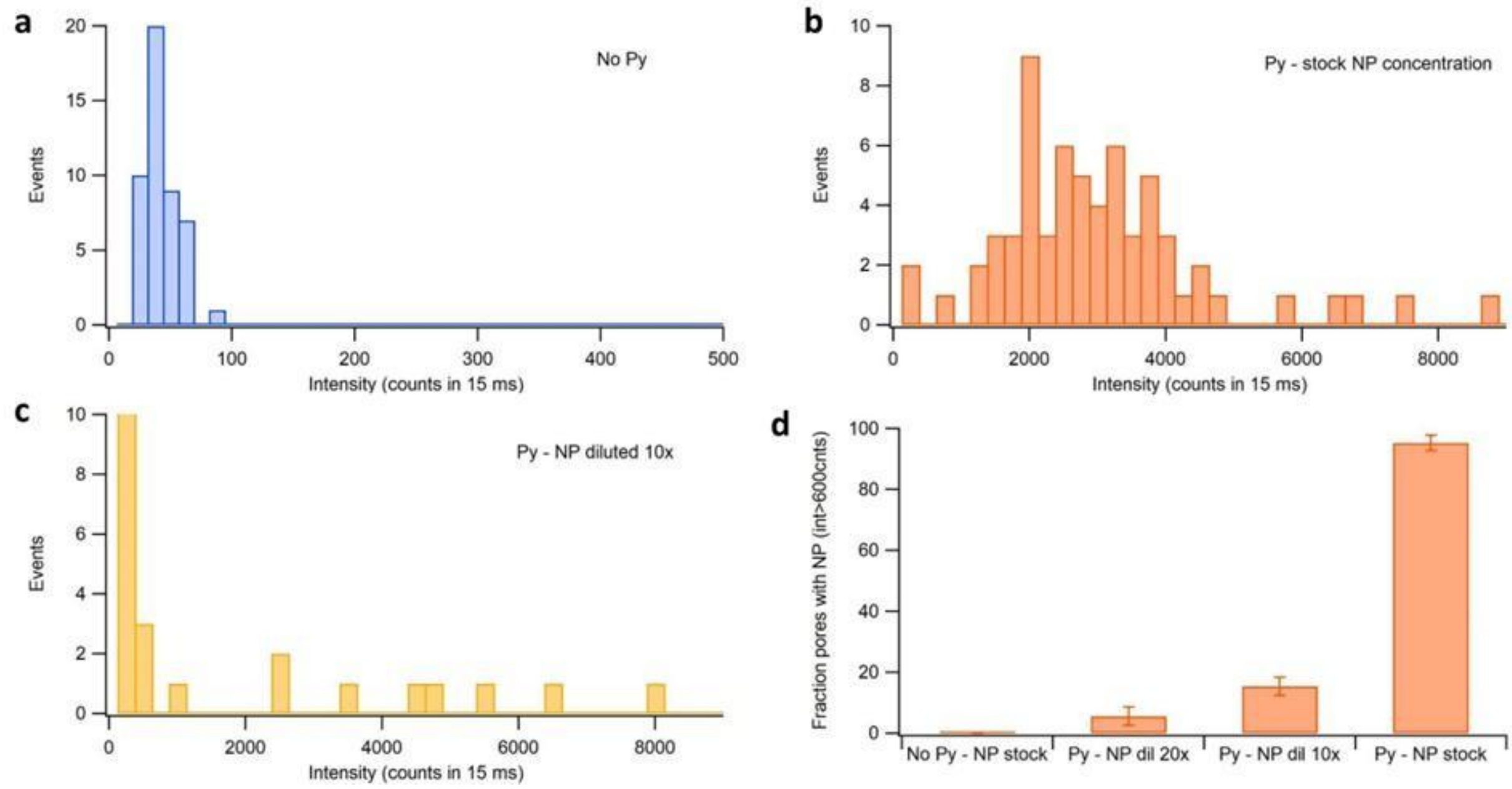

Figure S11. (a-c) distribution of the fluorescence intensities recorded for single nanopore in absence (a) or presence (b,c) of the Py layer. (d) Fraction of nanopores containing fluorescent NPs (defined using an intensity threshold of 600 counts/15ms) for different concentrations of the NP solution.
